# Network Meta-Analysis of the Efficacy of Acupuncture, Alpha-blockers and Antibiotics on Chronic Prostatitis/Chronic Pelvic Pain Syndrome

**DOI:** 10.1038/srep35737

**Published:** 2016-10-19

**Authors:** Zongshi Qin, Jiani Wu, Jinhui Tian, Jing Zhou, Yali Liu, Zhishun Liu

**Affiliations:** 1 Department of Acupuncture, Guang’anmen Hospital, China Academy of Chinese Medical Sciences, Beijing, China; 2 Beijing University of Chinese Medicine, Beijing, China; 3 Evidence-Based Medicine Center, School of Basic Medical Sciences, Lanzhou University, Lanzhou, China; 4 Institute of Basic Research in Clinical Medicine, China Academy of Chinese Medicine Sciences, Beijing, China

## Abstract

Alpha-blockers and antibiotics are most commonly used to treat chronic prostatitis/chronic pelvic pain syndrome (CP/CPPS) in clinical practice. Currently, increasing evidence also suggests acupuncture as an effective strategy. This network meta-analysis intended to assess the comparative efficacy and safety of acupuncture, alpha-blockers and antibiotics for CP/CPPS. Twelve trials involving 1203 participants were included. Based on decreases in the National Institutes of Health Chronic Prostatitis Symptom Index (NIH-CPSI) score, a network meta-analysis indicated that electro-acupuncture (standard mean difference [SMD]: 4.29; 95% credible interval [CrI], 1.96–6.65), acupuncture (SMD: 3.69; 95% CrI, 0.27–7.17), alpha-blockers (SMD: 1.85; 95% CrI, 1.07–2.64), antibiotics (SMD: 2.66; 95% CrI, 1.57–3.76), and dual therapy (SMD: 3.20; 95% CrI, 1.95–4.42) are superior to placebo in decreasing this score. Additionally, electro-acupuncture (SMD: 2.44; 95% CrI, 0.08–4.83) and dual therapy (SMD: 1.35; 95% CrI, 0.07–2.62) were more effective than alpha-blockers in decreasing the total NIH-CPSI total score. Other network meta-analyses did not show significant differences between interventions other placebo. The incidence of adverse events of acupuncture was relatively rare (5.4%) compared with placebo (17.1%), alpha-blockers (24.9%), antibiotics (31%) and dual therapy (48.6%). Overall, rank tests and safety analyses indicate that electro-acupuncture/acupuncture may be recommended for the treatment of CP/CPPS.

Chronic prostatitis/chronic pelvic pain syndrome (CP/CPPS) refers to National Institutes of Health (NIH) category III prostatitis, defined as urologic pain or discomfort in the pelvic region lasting for at least 3 months during the preceding 6 months that is associated with urinary symptoms, and not accompanied by a urinary tract bacterial infection^1^. CP/CPPS can impair both the physical and psychological function of patients and frequently diminishes their quality of life. It is the most common urological diagnosis among adult men under the age of 50 and accounts for 2 million office visits to urologists in the United States^2^,^3^. As the most common type of prostatitis, approximately 90% to 95% of men with symptoms of chronic prostatitis have CP/CPPS^4^. According to a survey conducted by Duloy and colleagues in 2007, the annual cost to treat prostatitis is approximately $84 million. Thus, prostatitis places a significant economic burden on both individuals and society attributed by causing productivity absenteeism and incurring health care cost^3^.

At present, a gold standard diagnostic test for CP/CPPS is not available because the aetiology of this disease is poorly understood. Thus, diagnosis typically based on exclusion^5^,^6^,^7^. In 1999, the National Institutes of Health Chronic Prostatitis Symptom Index (NIH-CPSI) was developed and has been widely used to rapidly assess the severity of CP/CPPS symptoms. This questionnaire covers the three most important symptom domains: evaluating pain, voiding and quality of life (QoL), which can provide an overall and valid assessment^8^.

Due to the lack of effective treatment for CP/CPPS, a wide range of therapies have been routinely used to treat this condition, including alpha-blockers, antibiotics, non-steroidal anti-inflammatory drugs (NSAIDs), and other agents^9^. Among these agents, alpha-blockers and antibiotics are most commonly used in clinical practice^10^,^11^. However, alpha-blockers and antibiotics exert only moderate, albeit significant, beneficial effects^1^. Moreover, adverse effects, such as dizziness, nausea, postural hypotension, and gastrointestinal complaints also reduce the patients’ compliance to therapy, which may affect the efficacy of treatment^1^,^6^.

In recent years, increasing evidence implicating acupuncture as a possible strategy for CP/CPPS treatment has accumulated^12^,^13^,^14^,^15^. A direct meta-analysis indicated that acupuncture is superior to sham acupuncture and partial drugs (levofloxacin, tamsulosin, and ibuprofen) in improving the symptoms of CP/CPPS^16^,^17^. However, a meta-analysis of direct comparisons between acupuncture and different classes of oral drugs is unavailable because such analyses are limited by the comparators and insufficient studies.

Network meta-analysis overcomes this limitation by creating indirect comparisons and allowing data synthesis, which could help identify the most effective interventions^18^,^19^. Therefore, we performed this Bayesian network meta-analysis and systematic review to discover both direct and indirect comparisons of acupuncture, alpha-blockers and antibiotics. To this end, we compared changes in the total NIH-CPSI score and three sub-domain scores. In addition, we also analysed the incidence rates of adverse events amongst included interventions.

## Results

### Study Selection

**Figure 1:**
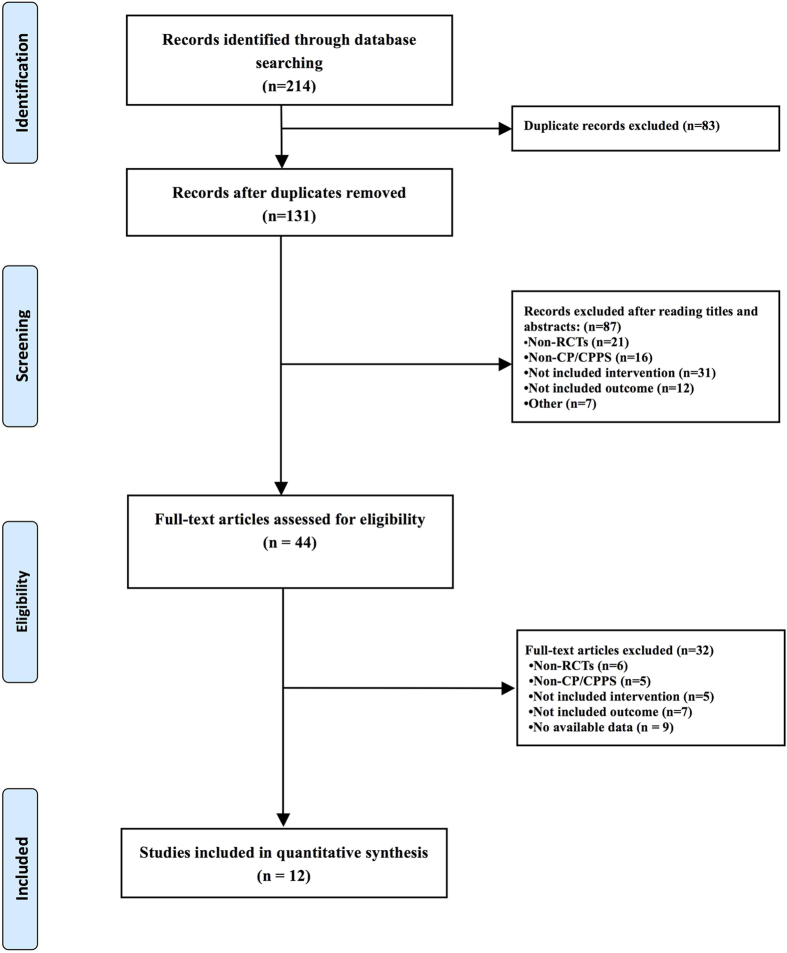
PRISMA flow chart

The search was performed on Feb 2^nd^, 2016 and identified 214 references. After duplicate studies were removed, another 87 records were excluded by reading the titles and abstracts, and the full texts of 44 articles were then assessed for eligibility. Finally, a total of 12 studies covering 7 groups, acupuncture, electro-acupuncture, alpha-blockers, antibiotics, dual therapy, sham acupuncture and placebo, were included^12^,^13^,^20^,^21^,^22^,^23^,^24^,^25^,^26^,^27^,^28^,^29^. The PRISMA flow chart of study selection is depicted in Fig. 1.

### Study Description

**Figure 2:**
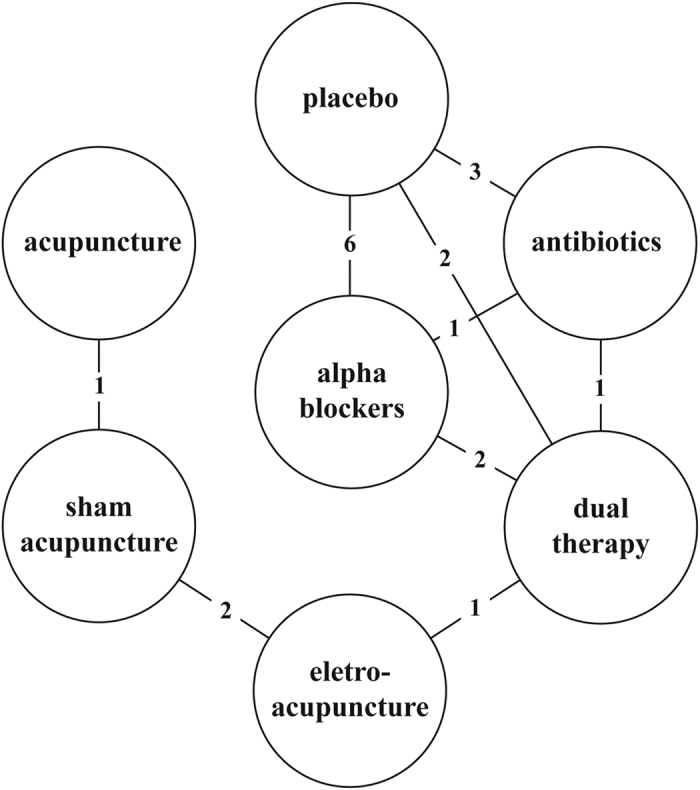
Network plot.

Three trials compared acupuncture or electro-acupuncture to sham acupuncture^12^,^20^,^27^, one trial compared electro-acupuncture to antibiotics combined with NSAIDs^13^, eight trials were pooled to compare placebo with alpha-blockers or antibiotics or their combination^21^,^22^,^23^,^24^,^25^,^26^,^28^,^29^. Overall, 1203 patients were included in the network meta-analysis. The participants were come from Canada^24^,^26^,^29^, the United States^21^,^23^, Turkey^12^,^13^,^22^, Korea^27^, Malaysia^20^,^21^,^24^, and China^26^,^29^. The characteristics of included studies are summarized in Table 1. All studies reported NIH-CPSI total scores and subdomain scores as outcomes. The network plot of eligible comparisons for NIH-CPSI scores is shown in Fig. 2, and the risk of bias of studies is summarized in Table 2. Furthermore, more than half of the included trials did not provide the details of their allocation concealment. The mean, SD, and sample size between treatment groups for studies included in the network meta-analysis are summarized in Supplementary Table 1.

### NIH-CPSI Total Score

Assessing primary outcome, the results of a pair-wise meta-analysis suggested that compared with sham acupuncture, acupuncture (SMD: 0.97; 95% CI, 0.17–1.78) and electro-acupuncture (SMD: 1.59; 95% CI, 0.65–2.53) resulted in significantly larger changes in the NIH-CPSI score than sham acupuncture. In addition, alpha-blockers (SMD: 1.38; 95% CI, 0.51–2.24) and antibiotics (SMD: 2.06; 95% CI, 0.18–3.96) significantly improved the NIH-CPSI score compared with placebo. The remaining 4 comparisons between other active treatments did not show the significant differences with the exception of electro-acupuncture, which was superior to dual therapy (SMD: 1.09; 95% CI, 0.52–1.67).

**Figure 3:**
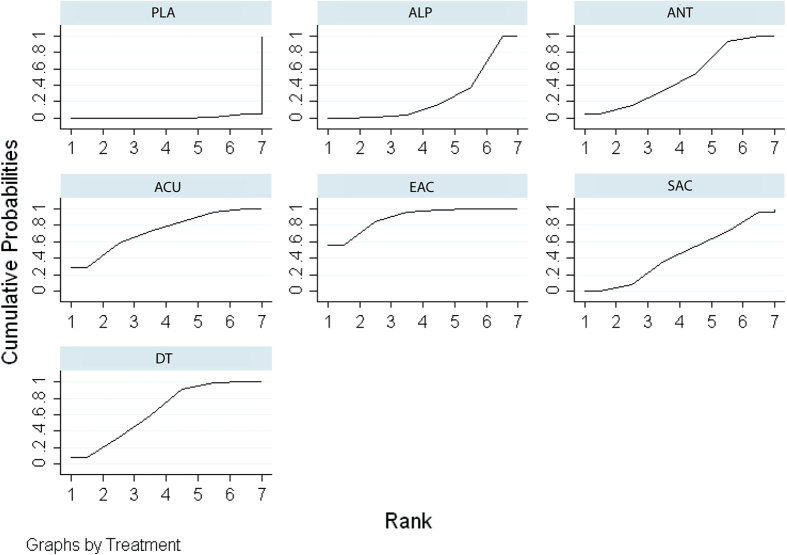
SUCRA for NIH-CPSI total score.

The network meta-analysis showed that all treatments other than sham acupuncture were more efficacious than placebo. Specifically, electro-acupuncture (SMD: 4.29; 95% CrI, 1.96–6.65), acupuncture (SMD: 3.69; 95% CrI, 0.27–7.17), alpha-blockers (SMD: 1.85; 95% CrI, 1.07–2.64), antibiotics (SMD: 2.66; 95% CrI, 1.57–3.76), and dual therapy (SMD: 3.20; 95% CrI, 1.95–4.42) were more efficacious than placebo, and network comparisons did not show no significant differences between the remaining pairs of indirect comparisons. The results of direct and indirect comparisons in the total NIH-CPSI score are shown in the lower and upper triangles of Table 3, and significant differences are underlined and bolded. The absolute effects and rank test indicated that electro-acupuncture was the most effective strategy in terms of reducing the total NIH-CPSI score, followed by acupuncture, dual therapy, antibiotics, sham acupuncture, alpha-blockers, and placebo. The surface under the cumulative ranking probabilities (SUCRA) of the total NIH-CPSI score is shown in Fig. 3.

### NIH-CPSI Pain Score

The pair-wise meta-analysis showed that electro-acupuncture was preferred to sham acupuncture (SMD: 1.88; 95% CI, 2.87–0.89) and dual therapy (SMD: 0.82; 95% CI, 1.38–0.26) to improve the NIH-CPSI pain domain score. Alpha-blockers were better than placebo (SMD: 1.05; 95% CI, 0.27–1.83) in improving this score. No significant difference was detected in the remaining 6 direct comparisons.

**Figure 4:**
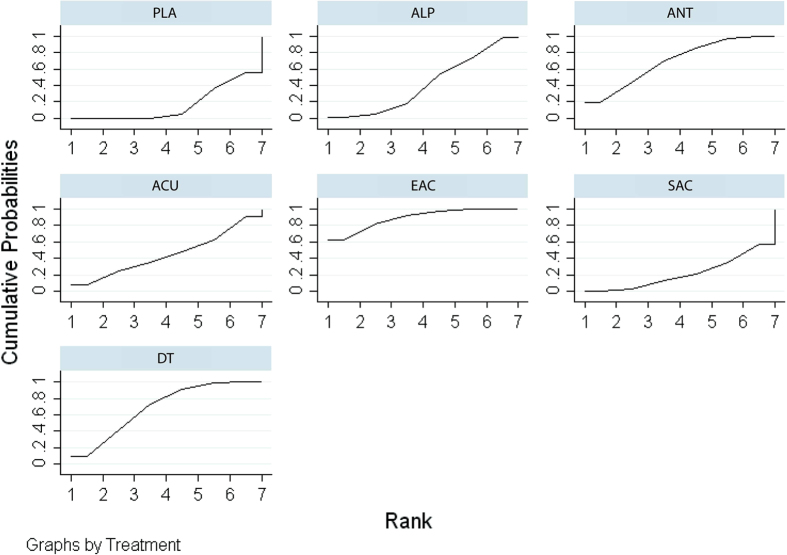
SUCRA for NIH-CPSI pain score.

The network meta-analysis indicated that electro-acupuncture (SMD: 2.30; 95% CrI, 0.03–4.63), dual therapy (SMD: 1.46; 95% CrI, 0.23–2.68), and antibiotics (SMD: 1.47; 95% CrI, 0.39–2.54), but not alpha-blocker, acupuncture, and sham acupuncture, were associated with a significantly higher improvement in pain relief than placebo. Compared with sham acupuncture, electro-acupuncture was better at relieving pain (SMD: 2.38; 95% CrI, 0.33–4.43). The remaining indirect comparisons did not show significant differences. The results of direct and indirect comparisons in the NIH-CPSI pain score are shown in lower and upper triangles of Table 4, and significant differences are underlined and bolded. The absolute effects and rank test indicated that electro-acupuncture ranked the first, followed by dual therapy, antibiotics, alpha-blockers, acupuncture, sham acupuncture, and placebo. The SUCRA of the NIH-CPSI pain score is shown in Fig. 4.

### NIH-CPSI Voiding Score

A pair wise meta-analysis suggested that acupuncture was associated with a significantly larger improvement in the NIH-CPSI voiding domain score than sham acupuncture (SMD: 0.83; 95% CI, 0.21–1.45). Alpha-blockers (SMD: 0.75; 95% CI, 0.18–1.32) and antibiotics (SMD: 2.05; 95% CI, 0.18–3.93) resulted in larger improvements than placebo. No significant differences were detected in the remaining 6 direct comparisons.

**Figure 5:**
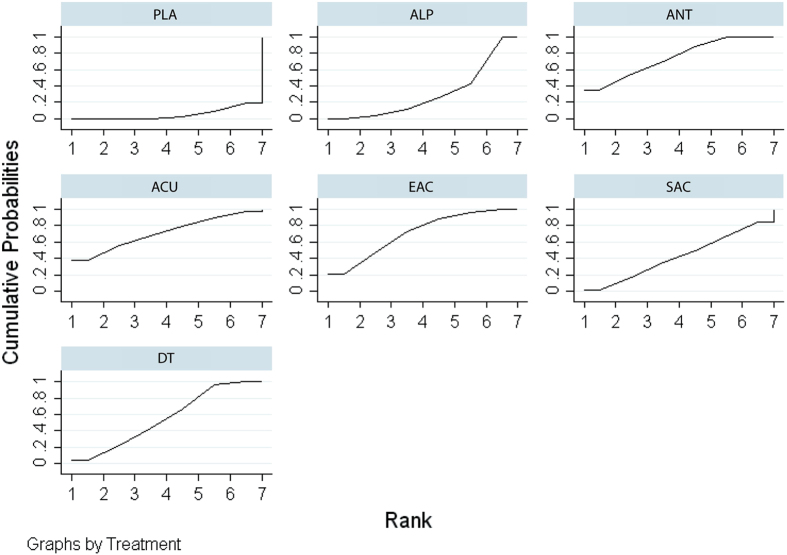
SUCRA for NIH-CPSI voiding score.

A network meta-analysis indicated that antibiotics (SMD: 2.25; 95% CrI, 1.20–3.31), dual therapy (SMD: 1.68; 95% CrI, 0.47–2.89), and alpha-blockers (SMD: 0.91; 95% CrI, 0.14–1.67), but not acupuncture, electro-acupuncture, and sham acupuncture, were associated with significantly larger improvements than placebo. Alpha-blockers provided better voiding relief than antibiotics (SMD: 1.34; 95% CrI, 0.13–2.55). None of the other remaining indirect comparisons showed significant differences. Direct and indirect comparisons of the NIH-CPSI voiding score are shown in the lower and upper triangles of Table 5, and significant differences are underlined and bolded. The absolute effects and rank test indicated that antibiotics ranked the first, followed by acupuncture, electro-acupuncture, dual therapy, sham acupuncture, alpha-blockers, and placebo. The SUCRA of the NIH-CPSI voiding score is shown in Fig. 5.

### NIH-CPSI QoL Score

A pair-wise meta-analysis showed that acupuncture was associated with a significantly larger improvement in the QoL than sham acupuncture (SMD: 1.10; 95% CI, 0.79–1.41), and alpha-blockers is superior to placebo in improving the QoL (SMD: 0.70; 95% CI, 0.09–1.31). The remaining 6 direct comparisons did not show significant differences.

**Figure 6:**
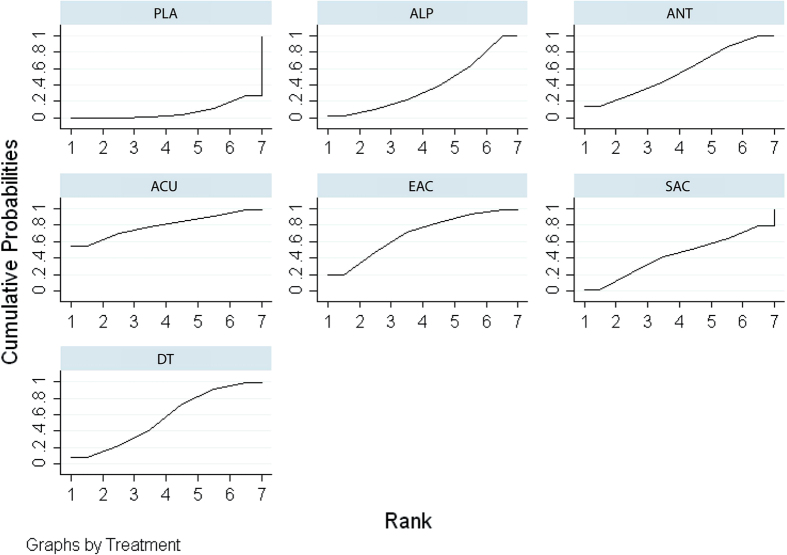
SUCRA for NIH-CPSI QoL score.

A network meta-analysis indicated that dual therapy (SMD: 1.25; 95% CrI, 0.07–2.41), antibiotics (SMD: 1.22; 95% CrI, 0.20–2.25) and alpha-blockers (SMD: 0.85; 95% CrI, 0.11–1.59) significantly improved the QoL domain score compared with placebo. Antibiotics were more effective than alpha-blockers (SMD: 0.38; 95% CrI, 0.81–1.55). Drect and indirect comparisons of the NIH-CPSI QoL score are shown in the lower and upper triangles of Table 6, and significant differences are underlined and bolded. The absolute effects and rank test indicated that acupuncture ranked the first, followed by electro-acupuncture, dual therapy, antibiotics, sham acupuncture, alpha-blockers, and placebo. The SUCRA of the NIH-CPSI QoL score is shown in Fig. 6.

### Safety

Eleven RCTs reported adverse events (AE)^12^,^13^,^20^,^21^,^22^,^23^,^24^,^25^,^27^,^28^,^29^. Dual therapy (DT) resulted in the highest incidence rate of AEs (48.6%), followed by antibiotics (31%), alpha-blockers (24.9%), and placebo (17.1%). Most of these AEs were moderate (e.g., dizziness, nausea, postural hypotension, and gastrointestinal complaints). Acupuncture was rarely associated with AEs (5.4%), which were generally mild (e.g., haematomas).

### Sensitivity Analysis and Network Assumption

The heterogeneity in the pair-wise meta-analysis was high among the three pairs for each outcome (placebo compared with alpha-blockers, antibiotics, and dual therapy). After a sensitive analysis, the sources were explored in three longest studies of alpha-blockers (24 weeks) and antibiotics (12 weeks)^22^,^26^,^29^. However, insufficient studies in the network prevented a meta-regression. In total, 4 loops were part of this network meta-analysis. The pair-wise meta-analysis and the network meta-analysis results did not significantly differ. Moreover, the inconsistency test did not show inconsistency between loops because their 95% CI included 0, as indicated by forest plots (inconsistency test are shown in Supplementary Figures 1–4).

## Discussion

The purpose of this systematic review and network meta-analysis was to identify the efficacy and safety of acupuncture and routine oral medications (alpha-blockers and antibiotics) for ameliorating the symptoms of CP/CPPS. Because dual therapy is commonly used in clinical practice, combinations of alpha blockers, antibiotics and NSAIDs were also included in the comparisons^30^. The assessment of the risk of bias (ROB) indicated that most included studies were characterized by a low ROB. Our primary analysis showed the following: although the direct and indirect comparison showed that all strategies except for sham acupuncture were associated with a significantly higher improvement in the total NIH-CPSI score than placebo, the results of indirect comparison of active strategies did not show significant differences between each active interventions. Ranking graphs of the primary outcome showed that electro-acupuncture was the most efficacious in improving the total NIH-CPSI score improvement, followed by acupuncture, dual therapy, antibiotics, sham acupuncture and alpha-blockers. Thus, both manual acupuncture and sham acupuncture are associated with improvement in outcomes, although an indirect comparison of these treatments did not show significant differences. Moreover, complex clinical devices seemed to have a stronger placebo effect than medication^31^; thus, acupuncture may associated with stronger placebo effect and expectation than medication. In terms of a safety analysis, acupuncture was associated with the lowest incidence of adverse events compared with alpha-blockers, antibiotics and dual therapy, and dual therapy was associated with the highest incidence of adverse events. In addition, for alpha-blockers and antibiotics, a longer treatment duration seemed to be more efficacious than short-term administration. These results might provide an overview of the efficacy and safety data for further clinical practice.

Because the aetiological factors of CP/CPPS remain poorly understood, treatment remains difficult. Based on clinical experience and mechanism-based reasoning, alpha-blockers and antibiotics are the two most commonly prescribed treatments prescribed by physicians. The bladder neck and prostate are rich in alpha-receptors, and alpha-receptors located in the central nervous system have been implicated in long-term pain syndromes. Recent preclinical data have suggested that alpha-blockers, such as alfuzosin, may reduce neurogenic inflammation in the lower urinary tract^32^,^33^. Moreover, alpha-blockers are commonly administered to men with BPH, which might have overlapping symptoms with CP/CPPS^34^,^35^. In addition to alpha-blockers, antibiotics such as quinolones and tetracycline are another treatment option for this disorder. Some studies have suggested that CP/CPPS is likely related to an infection with nanobacteria (NB), mainly because NB have been shown to cause multiple organic infections, especially urologic infection. However, NB are difficult to detected^36^,^37^. Nevertheless, antibiotics may be able to partially relieve the symptoms of patients with CP/CPPS because of possible underlying NB infection. With the exception of several Asian countries, acupuncture has not been used widely to treat CP/CPPS although its positive effects were initially demonstrated in 2008 by Lee in a randomized controlled trial^28^, and several randomized controlled trials were performed thereafter^12^,^13^,^27^. Acupuncture might may affect local peripheral events, spinal and central mechanisms or the combination thereof^15^. Furthermore, acupuncture regulates immune function, such as the cholinergic anti-inflammatory pathway, which may also be involved in inhibiting the inflammatory response^38^,^39^,^40^.

In 2011, Anothaisintawee and colleagues reported a network meta-analysis that compared mean symptom scores and treatment responses among oral managements for CP/CPPS, including alpha-blockers, antibiotics, anti-inflammatory drugs, and other active drugs^41^. This study suggested that alpha blockers, antibiotics and their combinations are appropriate strategies for the treatment of CP/CPPS. The results of our meta-analysis were consistent with the results concluded from the previous study. However, acupuncture was clearly more effective than not only placebo and sham acupuncture but also alpha blockers and antibiotics. Moreover, two pair-wise meta-analyses to assess the efficacy of acupuncture for CP/CPPS have recently been conducted^16^,^17^. The direct comparison indicated that acupuncture more effectively decrease the total NIH-CPSI score than sham acupuncture and standard medicine (levofloxacin, tamsulosin, and ibuprofen), and the evidence supported acupuncture as an effective treatment for CP/CPPS. Nonetheless, data on different classes of oral medication (alpha blocker, antibiotic, NSAIDs) were synthesized in the prior pair-wise meta-analysis due to limitations in methodology and the quantity of references, which limits the power of the aforementioned conclusion^16^,^17^. Multi-comparisons take advantage of indirect comparisons and provide a method to compare insufficient studies. In this network meta-analysis, acupuncture was compared with other routine strategies for men with CP/CPPS, and rank graphs based on absolute effects were provided.

The strengths of this study are as follows. We used a Bayesian framework to compare acupuncture with two mainstream active oral medications for CP/CPPS, and the results showed that acupuncture may be an efficacious and safe treatment for this condition. Although the results of the pooled indirect meta-analysis did not show significant differences between active treatments, an available ranking graph might be helpful for clinicians and further research. In addition, during the data synthesis, values that had been changed from baseline to final were used to arrive at a clinically worthwhile conclusion. Nevertheless, our study was also subject to limitations. Although 4 RCTs that assessed acupuncture as the control treatment, rendering direct comparison between acupuncture and oral drugs insufficient^12^,^15^,^21^. Additionally, half of the included studies reported changes from baseline to final^13^,^22^,^24^,^27^,^28^,^29^. In the remaining 7 studies, the changes in standard deviations were estimated with a R-value to equals to 0.5, which is a relatively conservative value and might enlarge the standard deviations compared with the originals values.

According to absolute effects and rank test, acupuncture/electro-acupuncture and dual therapy should be recommended to improve the total NIH-CPSI score. In addition, the evaluation of safety provides data to favour acupuncture. Due to the limitations in the quantity of currently available evidence, major direct comparisons were unavailable, and indirect comparisons between acupuncture and other active oral drugs did not show significant differences. High-quality randomized controlled trials with large numbers of participants that compare acupuncture to active medications should be conducted to explore the preferred options for clinical practice.

## Methods

This study was developed following the Preferred Reporting Items for Systematic Reviews and Meta-Analyses for Network Meta-Analysis (PRISMA-NMA) checklist^42^. (see Supplementary Table 2, which presents the PRISMA-NMA checklist).

### Study Selection

Two authors (JW and JZ) independently identified the eligible studies based on titles and abstracts. Full texts were scanned if a decision could not be made based on abstracts. Any disagreements in terms of study inclusion were resolved by discussion with a third party (ZL or ZQ).

### Eligibility Criteria

Randomized controlled trials that met following criteria were included: (1) Participants diagnosed with CP/CPPS (category III prostatitis according to NIH classification)^5^, but participants also suffering from benign prostatic hyperplasia (BPH) were excluded. (2) Trials comparing any pair of the following interventions: electro-acupuncture, acupuncture, alpha-blockers, antibiotics, combination of alpha-blockers or antibiotics or NSAIDs, sham acupuncture and placebo. Sham acupuncture was defined as invasive needle piercing into the sham acupoints, which did not correspond to any true acupuncture points. (3) Trials reporting one of following outcomes. The primary outcome for this study was the change in the total NIH-CPSI score from baseline to final treatment. The NIH-CPSI was developed to assess the symptoms and quality of life in men with CP/CPPS (with scores ranging 0–43 points, and higher scores indicating worse symptoms); it consists of three sub-scores: pain (0–21 points), urinary symptoms (0–10 points), and quality of life (0–12 points)^8^. Secondary outcomes included changes in the NIH-CPSI subscales from baseline to final treatment. In addition, adverse events due to the treatments were recorded.

### Data Sources and Searches

We performed electronic searches of following databases: Embase, PubMed, and the Cochrane Library. The search strategy consisted of three parts and included CP/CPPS (participants), alpha-blockers, antibiotics, acupuncture (interventions and controls), and a specific filter for randomized controlled trials (studies). The following keywords were used in combination with both MeSH terms and text words: chronic prostatitis, chronic pelvic pain, nonbacterial prostatitis, acupuncture, alpha blocker, alpha adrenergic receptor blocker, alpha adrenergic receptor antagonist, antibiotic, and antibacterial. No restriction were placed on language or publication status. In addition, to guarantee the saturation of literature, we also scanned relevant trials included by previous systematic reviews and meta-analyses for CP/CPPS as well. (see Supplementary Table 3, which describes the search terms and strategies).

### Data Collection Process

Four authors independently extracted and assessed the data (data extraction: ZQ and JW; assessment: JZ and YL) using a standard spread sheet (performed by Excel; Version 15.19.1), consisting of four sheets, which included (1) general information (i.e. study design, arms, intervention types, and information about data extractor); (2) study characteristic (i.e. patients, name of intervention, content of intervention, dosage and frequency, duration and follow-up, and outcomes); (3) a risk of bias assessment (ROB: randomization, allocation, blinding of participants and personnel, blinding of outcome assessor, incomplete data, selective reporting, and other bias); and (4) a summary of outcome data (dichotomous and continuous data).

For the studies that failed to report a before-and-after difference in outcome and instead reported the mean and standard deviation at baseline and at after treatment, the change from baseline was estimated using the methods recommended in the Cochrane Handbook^43^. Because information on R is seldom available, so we used 0.5 to estimate R, which is considered conservative^44^.

### Statistical Analysis

The pair-wise meta-analysis were initially performed to synthesize studies that compared the same interventions with random effects models (direct comparison) using the STATA software (Version 13.0; Stata Corporation, College Station, Texas, USA). Second, to determine comparative effectiveness, a random effects model network meta-analysis (combination of direct and indirect comparison) was developed in a Bayesian framework using Markov chain Monte Carlo simulation methods provided by the WinBUGS software (Version 1.4.3; MRC Biostatistics Unit, Cambridge, UK) with a Chaimani model^45^,^46^. The Markov chains were utilized for 50,000 simultaneous iterations based on the data and description of the proposed distributions for relevant parameters, of which, the first 10000 iterations were discarded because they may have an impact on the arbitrary value. The direct and indirect comparisons for each given pair of treatments were combined by modeling the continuous outcomes in every treatment group of enrolled studies. The NIH-CPSI score was reported as a standard mean difference (SMD) with a 95% confidence intervals (CI) for direct comparisons or 95% credible intervals (CrI) for indirect comparisons. In this process, the Brooks-Gelman-Rubin method was included to assess the convergence between direct and indirect variances^47^. To present the relationship among different treatments, we used a network plot to show the direct comparisons between arms. In addition, the effectiveness of each treatment among all available treatments was ranked by calculating the SMD in order^48^; plots of the SUCRA were generated by the STATA software^49^. Because the consistency among included trials is a basic principle used to conduct network meta-analyses, this result generated by an indirect comparison should be similar compared to the result derived from a direct comparison. We used the Z test to analyse the inconsistency of the model. A Z value and its corresponding p-value were calculated, and an R value less than 0.05 indicated a statistically significant difference^50^. The clinical, statistical, and methodological differences may be attributed to heterogeneity. The chi-squared test and I^2^ test were used to quantitatively assess heterogeneity quantitatively, and a p-value less than 0.1 in the chi-squared test or an I^2^ value statistic greater than 75% was considered significant. To identify the source of heterogeneity, sensitivity analyses were initially conducted by excluding trials with the longest or shortest duration of treatment. A meta-regression was not possible due to perform owing to the insufficient number of trials included.

## Additional Information

**How to cite this article**: Qin, Z. *et al.* Network Meta-Analysis of the Efficacy of Acupuncture, Alpha-blockers and Antibiotics on Chronic Prostatitis/Chronic Pelvic Pain Syndrome. *Sci. Rep.* **6**, 35737; doi: 10.1038/srep35737 (2016).

## Supplementary Material

Supplementary Information

## Figures and Tables

**Table 1:** Characteristics of Included Studies.

Study ID	Location	Sample size and intervention	Age*	Duration	Outcome	Adverse events
Lee^20^	Malaysia	44 acupuncture	40.9 ± 11.0	10 weeks	NIH-CPSI	8
		45 sham acupuncture	42.8 ± 9.4			5
Lee^27^	Korea	12 electro-acupuncture	39.8 ± 5.8	6 weeks	NIH-CPSI	0
		12 sham acupuncture	36.4 ± 5.8			1
Sahin^12^	Turkey	45 acupuncture	32.1 ± 7.2	6 weeks	NIH-CPSI	0
		46 sham acupuncture	32.8 ± 7.0			
Kucuk^13^	Turkey	26 electro-acupuncture	33.3 (17–50)	7 weeks	NIH-CPSI	0
		28 levofloxacin + ibuprofen				6 weeks
Cheah^24^	Malaysia	43 placebo	35 (20–50)	14 weeks	NIH-CPSI	11
		43 terazocin	36 (24–49)			21
Nickel^25^	Canada	45 placebo	56.2 (36–78)	6 weeks	NIH-CPSI	5
		35 levofloxacin	56.0 (39–77)			8
Alexander^23^	Canada/USA	45 placebo	42.6 ± 12.0	6 weeks	NIH-CPSI	20
		45 tamsulosin	45.3 ± 9.7			20
		42 ciprofloxacin	45.9 ± 11.7			19
		42 tamsulosin + ciprofloxacin	44.5 ± 11.4			17
Tugcu^22^	Turkey	29 placebo	29.1 ± 5.2	24 weeks	NIH-CPSI	6
		29 doxazosin	12			
		28 doxazosin + ibuprofen	17			
Nickel^21^	USA/Malaysia	138 placebo	40.1 ± 12.3	12 weeks	NIH-CPSI	7
		134 alfuzosin	40.1 ± 11.4			3
Zhou^26^	China	24 placebo	NR	12 weeks	NIH-CPSI	NR
		24 tetracycline				
Nickel^28^	Canada	54 placebo	49.0 ± 11.6	12 weeks	NIH-CPSI	7
		52 silodosin	49.2 ± 13.3			18
Chen^29^	China	47 placebo	33.3 ± 7.2	24 weeks	NIH-CPSI	2
		46 tamsulosin	35.3 ± 6.8			13

Summary of the included studies.

NIH-CPSI: National Institutes of Health Chronic Prostatitis Symptom Index; NR: not reported.

^*^Age is provided as the mean ± standard deviation or mean (range).

**Table 2:** Risk of Bias Assessment.

Study ID	Sequence generation	Allocation concealment	Blinding	Incomplete outcome	Selective outcome report	Other source of bias
Lee^20^	L	U	L	L	L	L
Lee^27^	L	U	L	L	L	H
Sahin^12^	L	U	L	L	L	L
Kucuk^13^	L	U	H	H	L	L
Cheah^24^	L	U	L	L	L	L
Nickel^25^	L	L	L	L	L	L
Alexander^23^	L	L	L	L	L	L
Tugcu^22^	U	U	L	L	L	L
Nickel^21^	L	L	L	L	L	L
Zhou^26^	U	U	U	U	L	H
Nickel^28^	L	U	L	L	L	L
Chen^29^	L	U	L	L	L	L

L: low risk of bias; H: high risk of bias; U: unclear.

Risk of bias assessment.

**Table 3:** Results of National Institutes of Health Chronic Prostatitis Symptom Index Total Score.

**PLA**	**1.85 (1.07, 2.64)**	**2.66 (1.57, 3.76)**	**3.69 (0.27, 7.17)**	**4.29 (1.96, 6.65)**	2.71 (−0.42, 5.88)	**3.20 (1.95, 4.42)**
**1.38 (0.52, 2.24)**	**ALP**	0.81 (−0.44, 2.05)	1.84 (−1.61, 5.31)	**2.44 (0.08, 4.83)**	0.86 (−2.29, 4.04)	**1.35 (0.07, 2.62)**
**2.06 (0.18, 3.96)**	0.26 (−0.16, 0.68)	**ANT**	1.03 (−2.49, 4.61)	1.63 (−0.86, 4.13)	0.05 (−3.16, 3.29)	0.54 (−0.95, 2.04)
NA	NA	NA	**ACU**	0.60 (−1.92, 3.09)	−0.98 (−2.38, 0.42)	−0.49 (−3.71, 2.72)
NA	NA	NA	NA	**EAC**	−1.58 (−3.67, 0.51)	−1.09 (−3.09, 0.90)
NA	NA	NA	**−0.97 (−1.78, −0.17)**	**−1.59 (−2.53, −0.65)**	**SAC**	−0.49 (−2.42, 3.37)
4.95 (−4.26, 13.45)	0.05 (−0.27, 0.37)	−0.31 (−0.73, 0.10)	NA	**−1.09 (−1.67, −0.52)**	NA	**DT**

PLA: placebo; ALP: alpha-blocker; ANT: antibiotic; ACU: acupuncture; EAC: electro-acupuncture; SAC: sham acupuncture; DT: dual therapy. NA: not available.

Results of direct and indirect comparisons in the total National Institutes of Health Chronic Prostatitis Symptom Index score.

**Table 4:** Results of National Institutes of Health Chronic Prostatitis Symptom Index Pain Score.

**PLA**	0.69 (−0.08, 1.47)	**1.47 (0.39, 2.54)**	0.61 (−2.79, 3.40)	**2.30 (−0.03, 4.63)**	−0.08 (−3.16, 3.02)	**1.46 (0.23, 2.68)**
**1.05 (0.27, 1.83)**	**ALP**	0.78 (−0.46, 2.00)	−0.08 (−3.49, 3.32)	1.61 (−0.74, 3.95)	−0.77 (−3.90, 2.34)	0.77 (−0.49, 2.03)
1.28 (−0.29, 2.85)	0.17 −0.25, 0.59	**ANT**	−0.86 (−4.36, 2.63)	0.83 (−1.63, 3.28)	−1.55 (−4.77, 1.64)	−0.01 (−1.48, 1.46)
NA	NA	NA	ACU	1.69 (−0.78, 4.15)	−0.69 (−2.07, 0.68)	0.85 (−2.29, 4.03)
NA	NA	NA	NA	**EAC**	**−2.38 (−4.43, −0.33)**	−0.83 (−2.81, 1.14)
NA	NA	NA	−0.69 (−1.63, 0.25)	**−1.88 (−2.87, −0.89)**	**SAC**	1.55 (−1.30, 4.39)
2.28 (−2.27, 6.67)	−0.02 (−0.35, 0.30)	−0.29 (−0.71, 0.14)	NA	**−0.82 (−1.38, −0.26)**	NA	**DT**

PLA: placebo; ALP: alpha-blocker; ANT: antibiotic; ACU: acupuncture; EAC: electro-acupuncture; SAC: sham acupuncture; DT: dual therapy. NA: not available.

Results of direct and indirect comparisons in National Institutes of Health Chronic Prostatitis Symptom Index pain score.

**Table 5:** Results of National Institutes of Health Chronic Prostatitis Symptom Index Voiding Score.

**PLA**	**0.91 (0.14, 1.67)**	**2.25 (1.20, 3.31)**	2.29 (−1.03, 5.59)	2.18 (−0.11, 4.46)	1.45 (−1.60, 4.47)	**1.68 (0.47, 2.89)**
**0.75 (0.18, 1.32)**	**ALP**	**1.34 (0.13, 2.55)**	1.38 (−1.94, 4.70)	1.27 (−1.02, 3.56)	0.54 (−2.52, 3.57)	0.77 (−0.46, 2.01)
**2.05 (0.18, 3.93)**	0.19 −0.23, 0.62	**ANT**	0.04 (−3.38, 3.44)	−0.07 (−2.49, 2.34)	−0.80 (−3.94, 2.32)	−0.57 (−2.01, 0.89)
NA	NA	NA	**ACU**	−0.11 (−2.54, 2.31)	−0.84 (−2.19, 0.52)	−0.61 (−3.70, 2.49)
NA	NA	NA	NA	**EAC**	−0.73 (−2.74, 1.28)	−0.50 (−2.44, 1.45)
NA	NA	NA	**−0.83 (−1.45, −0.21)**	−0.73 (−1.56, 0.10)	**SAC**	0.23 (−2.43, 3.03)
1.66 (−1.02, 4.34)	0.25 (−0.10, 0.59)	−0.1 (−0.53, 0.33)	NA	−0.49 (−1.03, 0.05)	NA	**DT**

PLA: placebo; ALP: alpha-blocker; ANT: antibiotic; ACU: acupuncture; EAC: electro-acupuncture; SAC: sham acupuncture; DT: dual therapy. NA: not available.

Results of direct and indirect comparisons in National Institutes of Health Chronic Prostatitis Symptom Index voiding score.

**Table 6:** Results of National Institutes of Health Chronic Prostatitis Symptom Index Quality of Life Score.

**PLA**	**0.85 (0.11, 1.59)**	**1.22 (0.20, 2.25)**	2.21 (−1.04, 5.47)	1.70 (−0.51, 3.93)	1.11 (−1.85, 4.08)	**1.25 (0.07, 2.41)**
**0.70 (0.09, 1.31)**	**ALP**	**0.38 (0.81, 1.55)**	1.36 (−1.90, 4.62)	0.86 (−1.38, 3.09)	0.26 (−2.71, 3.24)	0.40 (−0.80, 1.58)
1.00 (−0.17, 2.16)	0.27 −0.15, 0.69	**ANT**	1.00 (−2.34, 4.35)	0.48 (−1.86, 2.84)	−0.11 (−3.18, 2.96)	0.03 (−1.38, 1.42)
NA	NA	NA	**ACU**	−0.50 (−2.88, 1.87)	−1.10 (−2.41, 0.21)	−1.00 (−4.00, 2.07)
NA	NA	NA	NA	**EAC**	−0.60 (−2.57, 1.37)	−0.50 (−2.34, 1.43)
NA	NA	NA	**−1.10 (−1.41, −0.79)**	−0.59 (−1.41, 0.23)	**SAC**	0.14 (−2.59, 2.88)
1.77 (−1.63, 5.17)	−0.18 (−0.58, 0.23)	NA	NA	−0.46 (−1.00, 0.08)	NA	**DT**

PLA: placebo; ALP: alpha-blocker; ANT: antibiotic; ACU: acupuncture; EAC: electro-acupuncture; SAC: sham acupuncture; DT: dual therapy. NA: not available.

Results of direct and indirect comparisons in National Institutes of Health Chronic Prostatitis Symptom Index quality of life score.
